# Resource allocation decision-making in dementia care with and without budget constraints: a qualitative analysis

**DOI:** 10.12688/hrbopenres.13147.2

**Published:** 2020-12-14

**Authors:** Fiona Keogh, Tom Pierse, Eamon O'Shea, Christine Fitzgerald, David Challis

**Affiliations:** 1 Centre for Economic and Social Research on Dementia, National University of Ireland, Galway, Galway, Ireland; 2 Institute of Mental Health, University of Nottingham, Nottingham, UK

**Keywords:** Dementia, resource allocation, heuristic, context, health and social care

## Abstract

**Introduction**: Health systems in many different countries have increasingly been reorienting the delivery of dementia care to home and community care settings. This paper provides information on how health and social care professionals (HSCPs) in Ireland make decisions on resource allocation for people with dementia living at home and how resource constraints affect their decisions and choices.

**Methods:** A balance of care approach was used to assess resource allocation across six dementia case types, from low to high needs. Workshops were held with 24 HSCPs from multiple disciplines. Participants allocated services in two scenarios: allocation with and without a budget constraint. Nominal group technique was used to structure discussions around resource allocation in both scenarios. Thematic analysis was applied to analyse the qualitative data using a general inductive approach.

**Results: **The following themes influenced allocative deliberations: whose needs are being met; what needs are identified; decision making context; decision making process; and allocation outcomes. Participants were proficient in making decisions, using ‘decision rules’ or heuristics to help them make decisions under fixed budget rules and sticking to conventional provision when constraints were in place.

**Conclusions**: Freedom from a budget constraint allowed HSCPs to consider a broader range of services and to take a more expansive view on what needs should be considered, with a particular emphasis on adopting a proactive, preventative approach to the allocation of resources. The effect of the budget constraint overall was to narrow all considerations, using heuristics to limit the type of needs addressed and the range of services and supports provided. The consequences were a largely reactive, less personalised system of care. The findings emphasise the need for an integrated and comprehensive assessment process that is more concerned with individualised responses rather than relying on existing models of care alone.

## Introduction

For many years, health systems have been seeking to reorient the delivery of health and social care to home and community settings as much as possible, in an effort to improve outcomes, manage costs and achieve greater equity ( OECD, 2017; WHO, 2000). This is also the case in Ireland ( Oireachtas Committee on the Future of Healthcare, 2017)). The delivery of home and community based services is particularly important for people with dementia who, in common with many older people, have expressed a preference to remain living well at home for as long as possible ( Browne, 2016; McGee *et al.*, 2005). The care needs of people with dementia at the boundary between community and residential care have been widely studied using balance of care (BoC) methodology ( Tucker *et al.*, 2013). Less is known about decision making around care needs throughout the course of dementia, from early stages to more advanced dementia. In addition, there is little evidence on resource allocation decisions under budget constrained conditions in the context of home and community care for people with dementia.

Decision making in health care occurs at many different levels, from budget allocations for health and social care systems to care planning at the individual level. These levels have been labelled respectively as the macro-level and micro-level, with the organizational level in between labelled the meso-level ( Plochg & Klazinga, 2002). There is a significant amount of literature on macro-level decision making in health care, such as processes for allocating national or regional health budgets ( OECD, 2017; Ruane, 2010). The area of clinical decision making also has its own substantial literature, usually examining discipline-specific decision making in acute health care contexts ( Standing, 2010; Thokala *et al.*, 2016). In practice, most health and social care professionals (HSCPs) have a limited role in explicit resource allocation, typically working within budgets that have been allocated in a ‘top-down’ way from national to a local level. This may explain the absence of literature examining the role of HSCPs in resource allocation. However, almost all clinical decision making has resource implications and can be construed as resource allocation on a micro-level. It is this clinical and allocation decision making at the micro-level, that aggregates eventually to resource allocation at the macro level.

However, there is very little examination of micro-level decision making in community-based health and social care settings or at the meso-level, lying- between the micro level – how many home care hours should be provided to this person?; and the macro – what proportion of the national health budget should be allocated to home care? There is also little understanding of how this occurs for people with complex conditions such as dementia, who require a wide range of services and supports over a protracted period of time, and who are largely supported at home by family carers ( O’Shea & Monaghan, 2017). In common with most other jurisdictions, micro- and meso-level decision making in health care in Ireland occurs in conditions of significant resource constraints ( Brick *et al.*, 2010), but there is little empirical evidence on how budget constraints shape decision making or how these decisions impact on dementia care.

This study takes a ‘bottom-up’ approach to understanding resource allocation decision making in dementia care among HSCPs. We examine micro and meso-level decision making at the intersection of clinical decision-making and resource allocation decision making. This is done in two scenarios – decision-making under a fixed budget constraint and decision-making under no budget constraint. Decisions by HSCPs have significant implications for the quality of life of the person with dementia and their family carer, such as what activities the person can engage in, how long they can remain at home, and how well the carer is supported. These decisions have both efficiency and equity implications. Efficiency is concerned with linking costs and outcomes to produce maximum benefits to care recipients - how many hours? what type of hours? to what end? Equity is more concerned with ensuring that resources are allocated fairly, to those with the greatest level of need, irrespective of income, class or geography.

Resource allocation from the macro to the micro level is dependent on an assessment of need, which is itself a complex and often contested concept ( Asadi-Lari *et al.*, 2003; Dean, 2010). Maslow’s hierarchy of needs has been very influential, with its enduring concept of inter-related needs where one need is dependent on the fulfilment of a previous need ( Maslow, 1943). Bradshaw’s (1972) taxonomy defines four types of need; normative need - as defined by experts, often using standardised assessments; comparative needs – comparing the needs of different individuals or groups based on objective metrics; expressed need – what people demand but often measured in terms of what services people use or waiting lists (both imperfect measures); and felt need – an individual’s expression of their needs. Although very influential, this conceptualisation has raised much argument regarding who is best placed to define need: service users or professionals ( Dean, 2010). In this study, we focused on health and social care needs. The overall study included HSCPs, people with dementia and carers and therefore included normative and felt needs. This paper reports on decision making by HSCPs.

A mixed methods study was designed to address some of these identified gaps in the literature. The main objectives of the study were: to gain a greater understanding of the resource allocation decision making process among HSCPs; and to identify differences in decisions relating to dementia care in two scenarios – with a fixed budget constraint and with no budget constraint; with the aim of informing resource allocation for dementia services in Ireland. We collected quantitative data on resource allocation by HSCPs supplemented by qualitative data to elucidate the decision-making process. The focus in this paper is on the latter – understanding how HSCPs make resource allocation decisions. Other papers are in preparation to report on the quantitative findings and to report on a detailed comparison of the quantitative and qualitative data across three groups in the study: HSCPs, people with dementia and carers.

## Methods

The qualitative data presented here was collected from HSCPs participating within a broader mixed methods study. The materials and methods for the overall study are described here, as they were used in the production of the qualitative data.

### Participants

Senior managers in four regional health organisations were asked to identify HSCPs from a range of disciplines that had direct experience of working with people with dementia or allocating services to people with dementia living at home. An information sheet describing the study was sent to these individuals, along with an invitation to participate in one of five workshops that were organised around the country. Twenty-nine HSCPs were invited to participate. Five could not attend workshops due to scheduling conflicts. Twenty four attended, including; public health nurses (PHNs) (n=6), social workers (n=3), occupational therapists (OT) (n=2), physiotherapists (n=1), speech and language therapists (SLT) (n=1), dieticians (n=1), psychologists (n=1), mental health nurses (n=2), home care coordinators (n=4) and older person’s service managers (n=3). Participants were recruited from different therapeutic backgrounds, experience and location to give as much variety to the decision-making process as possible. While some participants may have known each other informally, they had not worked collaboratively as team members prior to the resource allocation exercise. Participants were primarily from community based primary care, social care or psychiatry of old age teams. Two participants were hospital based medical social workers. There were seven participants in CHO 2, five in CHO 3, four in CHO8 and eight in CHO9, with a mix of disciplines from each CHO.

### Research design

**Figure 1.**
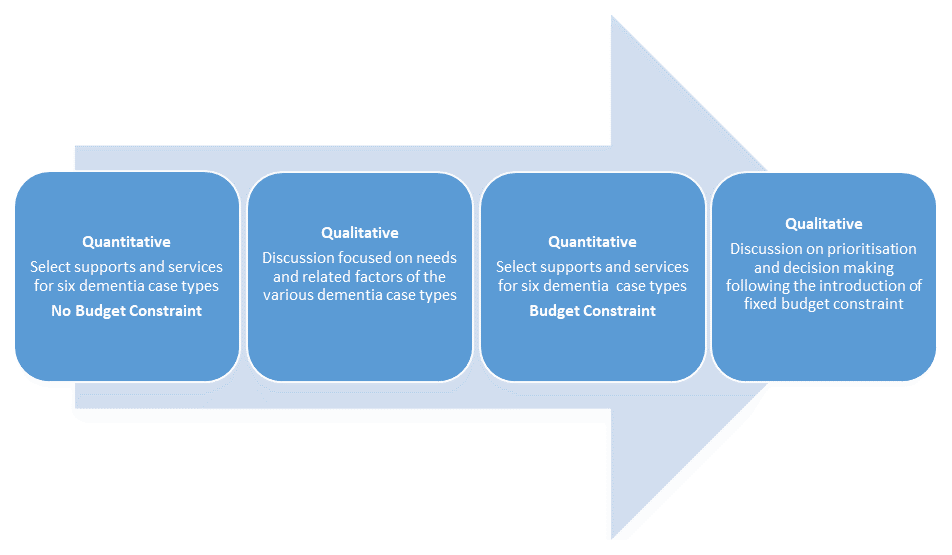
Research design.

The study used an explanatory sequential design with qualitative phases following on from quantitative phases as shown in Figure 1 ( Fetters & Freshwater, 2015).

Nominal group technique (NGT) was used to structure the quantitative exercises and qualitative discussion in five workshops, each with a multidisciplinary mix of HSCPs. The NGT method is used for exploring healthcare priorities and facilitates equal participation ( McMillan *et al.*, 2014). It typically consists of four phases: silent generation, round robin, clarification and ranking ( McMillan *et al.*, 2014). The materials used were vignettes to illustrate six case types and a service list.

### Development of case types and vignettes

Case types were specifically developed for this study using the approach adopted by Challis *et al.* (2014). Six dementia case types were developed which represented 46% of dementia cases in an Irish data set of 277 people with dementia ( O’Brien *et al.*, 2019), supplemented with data from SAT assessments of 453 inpatients over 65 ( Health Service Executive, 2017). Table 1 shows the different attributes for each case type. Vignettes were developed for each case type to lend realism and to help participants consider the needs of each case in allocating services. Study participants reported that each of the dementia case types used in the study was credible. A sample vignette is shown in Box 1.

**Table 1.** Variables and data sets used in the development of the case types.

	Data set: Assessments for home care service for people with dementia (N=277)							Variables derived from Single Assessment Tool data (N=453 inpatients) and literature			
Case Type	Dependency (Low, Medium, High)	Falls risk (Y/N)	Communication difficulty (Y/N)	Living alone (Y/N)	% of cases	Age	Sex (M/F)	Cognition (Mild, Moderate, Severe)	BPSD	Comorbidity	Amount of informal support (Low, Medium, High)
1	Low	N	N	N	9.5	84	F	Mild	None	Hypertension, Diabetes	Low
2	Medium	N	N	N	5.0	79	M	Mild	Depression and anxiety	Coronary Heart Disease	Medium
3	Medium	N	N	Y	9.5	82	F	Moderate	Irritability and persecution	Hypertension	Medium
4	Medium	Y	N	Y	9.9	86	M	Moderate	Wandering and hallucinations	None	Low
5	Medium	Y	Y	Y	3.4	83	F	Severe	Apathy and sleeping problems	Stroke	Medium
6	High	Y	Y	N	8.4	80	F	Severe	Sleep problems and psychotic symptoms	Hearing loss	High

BPSD, Behavioural and psychological symptoms of dementia.

Box 1. **Vignette 4 ‘Mr Dunne’**

**Home situation:** Mr Dunne is 86 years old and lives alone on a farm in a rural area. The house is heated by an open fire and is in a poor state of repair.

**Activities of daily living:** Mr Dunne needs help with eating, dressing and bathing. He often gets confused when dressing and puts his clothes on back to front and can forget to button up his shirt correctly. He has trouble getting in and out of the shower and needs to be reminded to wash. He has no issues with continence or in getting around the house.

**Cognitive impairment:** Mr Dunne’s short-term memory and concentration are moderately impaired. These difficulties were first recognised four years ago.

**Physical and mental health:** Mr Dunne can forget he is not as mobile as he used to be and has had several falls in the past year but no serious injury. He regularly walks into the village which is some distance away and on a busy road and someone has to drop him home. He has hallucinations periodically which he finds confusing and distressing.

**Informal support:** Mr Dunne is supported by his daughter who lives nearby. She spends several hours with Mr Dunne every day. She worries a lot about how they are going to cope in the future. She is taking medication for anxiety and depression and has a young family.

**Care preferences:** Mr Dunne is happy living at home but his daughter wants him to move to a nursing home.

### Service types

Participants were asked to allocate services as appropriate to needs across the six dementia case types. They were given a service list which was based on that used by Giebel *et al.* (2016) modified for the Irish context and informed by a mapping study of dementia-specific services in Ireland carried out in 2016/17 ( Alzheimer Society of Ireland & National Dementia Office, 2017). Twenty community-based service types were listed (see Table 2). Although all services in the list are not universally available throughout Ireland, all services listed are provided in at least one location. No gaps in the service list were noted by participants.

**Table 2.** Service List.

Service
Home Care
In-home Respite/Sitting Service (e.g. visiting service)
Re-ablement / Dementia support worker
Day Care (Standard or Dementia Specific)
Alzheimer’s Café, Dementia Social Clubs, or other support group for people with dementia
Dementia Friendly Activities
Meals on Wheels
Transport
Dementia Advisor
Carer Education Programme
Dementia Carer Support Groups
Counselling for family carer
Dementia Cognitive Therapies
Public Health Nurse
Specialist Dementia/Case Management
Day Hospital (Primary Care Centre)
Physiotherapist
Occupational therapist
Other Primary Care (Speech and Language/Dietician/Hearing)
Aids and Appliances (basic)
Referral to Psychiatry of Old Age Team
Nursing home based respite
Nursing Home Bed

### Workshops

A three-hour workshop was designed to collect the qualitative and quantitative data. The workshops were held in HSE facilities (usually administrative headquarters) and were facilitated by two of the authors (Fiona Keogh and Tom Pierse) one of whom has experience in running groups (FK). Two exercises were run: one with no budget constraint (NBC) and one with a budget constraint (BC). The six vignettes, the service list and service definitions were provided to participants. Each participant was provided with a computer, pre-loaded with a specially developed spreadsheet that showed the list of services that could be allocated for each dementia case type. Unit costs were embedded in the spreadsheet, but were hidden for the first NBC exercise. Data from a recent national audit of services used by people with dementia in Ireland was used to derive the monthly budget constraint ( Keogh *et al.*, 2020). Five workshops were held and each was audio recorded.

**NBC scenario:** In this scenario, participants were asked to allocate the type and amount of services that would be of most benefit to the person and carer in each of the six vignettes without considering budget constraints. This constituted the ‘silent phase’ of the NGT. Participants in the workshop then in turn presented to the group their allocation rationale for one case type, focusing on the needs they were trying to address through the service allocation. This constituted the ‘round robin’ phase of the NGT.

**BC scenario:** In this scenario, the costs of the services allocated in the first scenario for each case type were revealed. Participants were instructed to do the same exercise again but to work within an overall budget constraint of €7,000 to allocate care for all six dementia case types for one month. They could allocate this budget in any way they wished across the cases. Although participants in the first four workshops felt that this level of expenditure approximately reflected the current availability of resources, many found it difficult to stay within this constraint and tended to ‘overspend’. It was not feasible to enforce the constraint rigidly in the first three workshops and the average budget *de facto* expanded to €8,928, 28% above the initial constraint. For the final workshop, the budget was increased to €10,000 per month across the six dementia case types to explore whether a more relaxed constraint made the exercise easier for participants to complete.

In the BC scenario, time was allocated for discussion on which services participants cut in order to meet the budget constraint, and why, with an emphasis on articulating their decision making process. This was the NGT ‘clarification phase’. Finally, the average cost allocated by participants per dementia case type was calculated and displayed and the group discussed whether they agreed with the overall allocation of the budget per case type or if they wanted to make any changes after seeing the various relativities. This constituted a consensus check for the NGT, which allowed participants to review their choices. Brief follow up telephone or face-to-face interviews were conducted with 13 participants by FK and TP to gather further information on the decision-making process. The follow-up interviews were conducted to obtain contextual and technical information about local and/or discipline-specific resource allocation processes so that the authors had a greater understanding of the detailed discussions in the workshops. The participants were selected on the basis of being from a wide range of disciplines and possessing good technical knowledge of how the processes worked. All of the data reported here is from the workshops. The data from the interviews allowed a deeper understanding of the workshop data for the authors. These took, on average, 20 minutes and recordings and field notes were made.

### Qualitative data analysis

All recordings were transcribed and uploaded to NVivo version 12. The number of HSCPs in the workshops ranged from four to eight. It was not possible in the transcription process to separately identify each participant or their discipline each time they spoke. Although quotations presented here were drawn from a range of participants in all of the workshops, the quotations simply identify the CHO area. Thematic analysis can be a particularly useful approach for research applied to practice and policy ( Braun & Clarke, 2006). We applied the six-phase method of thematic analysis ( Braun & Clarke, 2006) to analyse the data using a general inductive approach ( Thomas, 2006). The design of the study resulted in qualitative data that was quite structured and the general inductive approach allowed us to identify the core meanings relevant to the research objectives.

**Figure 2.**
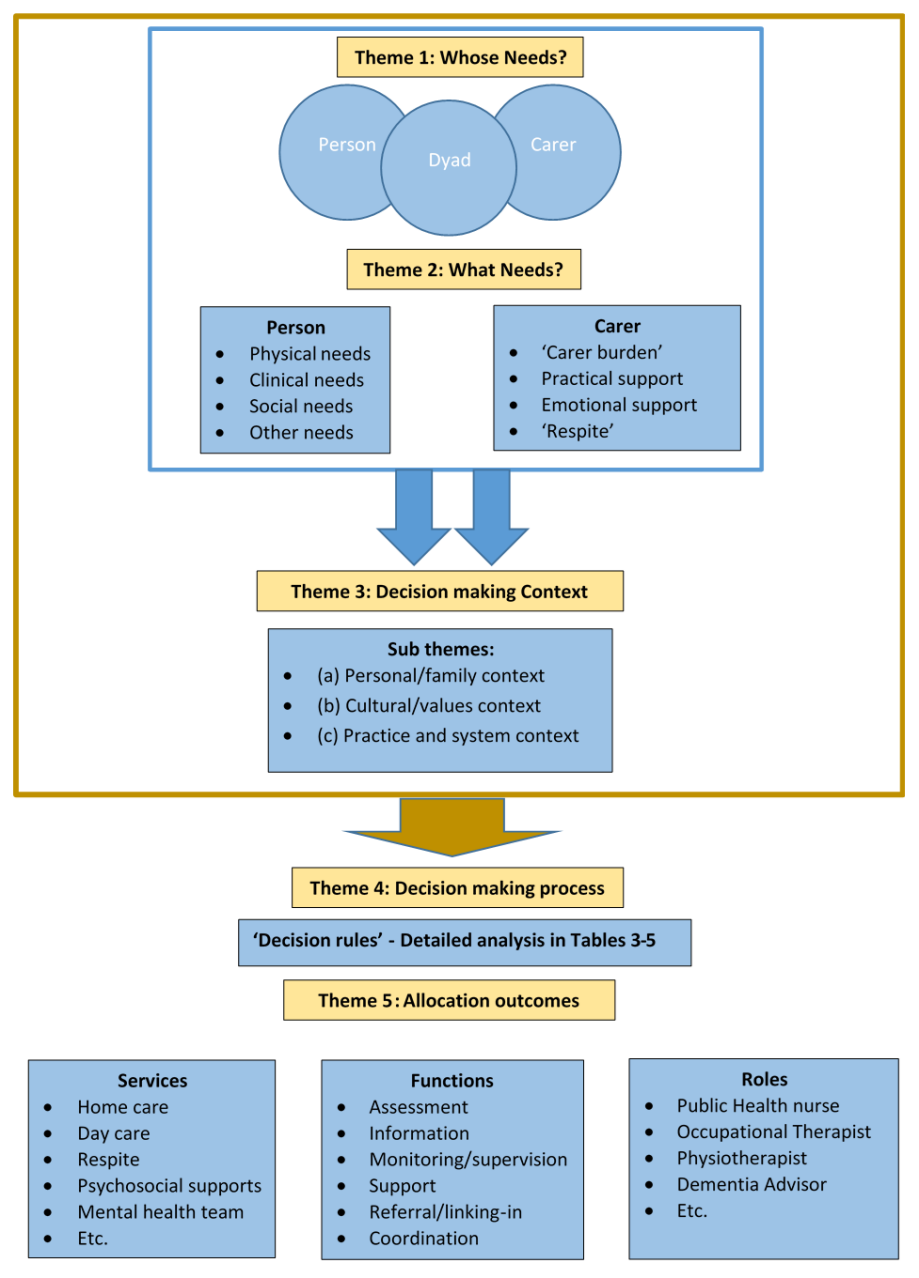
Thematic framework.

The coding process followed that outlined by Thomas (2006). Transcripts were read several times to become familiar with the data and to begin the preliminary identification of themes and categories. Three members of the team (FK, TP and CF) all coded the transcript from one workshop and compared coding to ensure consistency and to develop the coding frame for the other transcripts. All transcripts were coded in full by one of the research team (TP). Coding categories were considered in an iterative series of discussions and initial themes were identified. This resulted in a more manageable number of categories grouped into five main themes. A thematic framework ( Figure 2) was developed to summarise the themes and main categories and to show how these related to each other.

In order to explore the effect of the resource constraint on decision making in allocating resources, further examination of relationships within data across the themes was conducted to identify decision rules or heuristics that were used by the HSCPs when faced with the budget constraint. Heuristics are simple strategies or mental processes and rules that people use to form judgments quickly, make decisions, and find solutions to complex problems. This happens when an individual focuses on the most relevant aspects of a problem or situation to formulate a solution ( Gigerenzer & Gassmeier, 2011; Marewski & Gigerenzer, 2012).

The consolidated criteria for reporting qualitative research (COREQ) ( Tong *et al.*, 2007) were used to report and write up our analysis and the checklist has been uploaded (see Data availability section for details).

### Ethics

Ethical approval for the study was granted by the Research Ethics Committee of the National University of Ireland, Galway (REC 18-Jan-09). Information sheets were given to all participants in advance of the workshops and again at the workshops, with opportunities to ask questions in advance and at the beginning of the workshops. Written consent was obtained from all participants for participation in the study and use of anonymised data.

### Stakeholder engagement, public and patient participation (PPI)

As part of an applied partnership study, the research questions were identified in collaboration with senior managers from the National Dementia Office and national managers for older person’s services in Ireland. This partnership study also involved the Alzheimer Society of Ireland and an NGO supporting service reform, Genio.

The study used public and patient involvement (PPI) methods to involve people with dementia. A person with dementia was a member of the Oversight Group for this study, with input into study design, methods and measures. The list of services and case type vignettes were developed in consultation with two further people with dementia and two carers and modifications were made to both as a result.

## Findings

Five main themes were identified from the analysis of the qualitative data:

- Theme 1 – Whose needs are being met?
- Theme 2 – What needs are identified?
- Theme 3 – Decision making context
  - Sub-theme (a) Personal/family context
  - Sub-theme (b) Cultural/values context
  - Sub-theme (c) Practice and system context
- Theme 4 – Decision making process
- Theme 5 – Allocation outcomes

The framework in Figure 2 shows the relationships between themes and how some are ‘nested’ within others. When decisions were being made, the consideration of *whose needs* and *what needs* was ‘nested’ within a consideration of context, which included three types of context: personal, cultural and practice. Information from all three themes was brought to bear in theme 4, the *decision making process*. This theme is examined in detail in the analysis presented in Table 3– Table 5. The decision making process then results in allocation outcomes (theme 5) which includes not just services, but the identification of key roles and functions.

## Theme 1: Whose needs are being met?

A recurring theme in the discussions throughout the workshops related to whose needs were being considered in terms of service allocation. The discussions revealed a lack of clarity as to whose needs were being met and whose needs were a priority: whether the person, the carer or the dyad. HSCPs were clear on supporting the person with dementia to live well at home. Their wish to support the carer was also strongly expressed along with the rationale for doing this:

“it was about getting him enough support so that he can maintain his independence at home without putting a huge care burden on the wife and the daughter so that they don’t burn out, so that it can be sustained for a long period of time.” (CHO8)

When the budget constraint was introduced and allocations were pared back to meet the constraint, many HSCPs focused upon the person’s personal care needs, sometimes as a way of alleviating carer burden:

“I prioritised physical care needs, I suppose that was because there’s no point in someone having a day centre if they can’t get out and about.” (CHO2)

HSCPs found it difficult to disentangle the dyadic nature of benefits, focusing more on joint benefits rather than trade-offs between the parties. Participants noted that even a pared-back provision focusing on personal care for the person would be of some benefit to the carer in relieving some tasks:

“Because he can't dress himself, I just felt he does need a bit of a hand every morning,…and evening, it takes the pressure off his wife, that particular task.” (CHO9)

## Theme 2: What needs are identified?

The case vignettes described a number of needs (e.g. person needs help dressing) that were discussed by participants, leading to a more detailed articulation of needs for both the person with dementia and the carer.

### Needs of persons with dementia

Participants in the three groups identified a wide range of needs for people with dementia that are summarised here under four headings:

*Social*: leisure or activity of interest; social activity and social connection; getting out; structure in the day; and peer support, information and advice;
*Clinical/medical*: behavioural symptoms; nursing needs; medication; and supervision;
*Physical*: physical dependency and managing activities of daily living (ADLs and IADLs); falls prevention and rehabilitation; and maintaining independence and ability;
*Other*: Safety; personhood; adapting home; and changing needs and progression;

and neatly summarised in this quote:

“I suppose there’s the physical and nursing needs around and kind of her day to day needs, but also that she’s moderate cognitive impairment, but trying to keep her engaged as much as possible and try to keep her as independent as possible.” (CHO3)

### Needs of carers

HSCPs strongly emphasised the need to support family carers and frequently referred to carer burden as a ‘need’, although they did not always disentangle the different aspects of ‘burden’.

“There is a huge burden of care really on his family, which … wouldn’t be sustainable long term. So to try and prevent family burn out, it would be better to initiate home support services earlier, rather than later. (CHO3)

Of note in the discussions relating to the needs of both the person and the carer was the frequent conflation of needs with service responses, as in ‘this person needs day care’; ‘this family needs a respite service’ or ‘the PHN should be going in there’. There was often a lack of specificity as to what particular needs day care, respite or the PHN would be meeting.

For all cases, particularly the two with lower needs (cases 1 and 2 in Table 1), the discussion centred around the importance of being proactive, linking people with services and the potential of preventing or delaying problems. For the cases with higher needs (cases 4, 5 and 6), the discussion also covered the possibility of nursing home admission, particularly when the level of service required was described as ‘24/7’. Overall, though, there was a strong emphasis among participants on supporting the person to remain at home and to support the carers in their role.

The BC condition did not necessarily result in a different articulation of needs, as participants recognised that needs remained the same regardless of resources. However, the response to those needs and the prioritisation of needs was quite different under the BC. Participants focused on covering personal care and clinical needs above other needs:

“Quality of life is what I got rid of”. (CHO8).

The effect of the constraint is analysed in more detail in Table 4.

## Theme 3: Decision making context

It was clear from the data that decisions about needs and how best to meet them were intertwined with a consideration of multiple contexts. Three contexts were evident and were coded as sub-themes: (a) personal/family context; (b) cultural/values context; and (c) the practice/ system context.

### Sub-theme (a) Personal/family context

In this context, HSCPs were taking into account practical issues such as the availability of carers, the preferences of people with dementia and carers and the living environment. For example, in assessing the level of carer burden and trying to calibrate the amount of service to meet the need, a very complex weighing-up of different variables was occurring, such as the number of carers, the demands on their time, their age, their health problems, their expectations and family dynamics:

“…they’re gone, working, they’re gone earlier, they have child-minding, they have all the expenses.” (CHO2)

In spite of the difficulties for carers and families, participants acknowledged the enormous support provided by most families:

“And it’s amazing really, you know, how much families do, they actually are doing, they are doing so much and all they’re asking for is just that little bit of help with maybe the personal care or a bit of in home respite or, you know.” (CHO2)

HSCPs recognised preferences, autonomy and personhood (in various guises). Although these issues were not dominant in discussions, they came to the fore when difficult decisions were to be made, such decisions surrounding nursing home admission:

“…he needs independence but what's his [decision making] capacity, that’s the big thing. If he wants to stay at home, he can't change that.” (CHO9)

Issues such as whether the person lived alone; how far away the primary carer lived; the physical condition of the home; money and finances; accessibility of services in a rural area were considered. For example:

“The bit that’s sometimes forgotten with home supports is the condition of the house and the implications for the workers because the home has become a place of work… One of the main reasons we suspend care is safe environment.” (CHO9)

### Sub-theme (b) Cultural/values context

The discussions of HSCPs about the practical considerations of the personal/family context revealed that their decision-making is driven in part by the cultural context and the narratives and values they hold. Assumptions and generalisations were made about what families do and what families ought to be doing, which can also influence the perception of needs and allocation of care services. For example:

“I mean maybe those of that are a certain vintage, maybe have come up with the idea we looked after granny and granny was at home, and all that. But I notice the younger cohort… they’re not interested in doing incontinence, and all that kind of thing is “Oh no”. It’s a different, I think, mentality. “I shouldn’t be expected to do that.” (CHO2)
“…can have daughters living next door and they won’t go in and see their parents. We have daughters living 30, 40 kilometres, … and they’re going up and down every day. …Families are complicated.” (CHO3)

Attitudes of HSCPs to risk and capacity, and the difficulties that arise when balancing risk against the autonomy and agency of people with dementia also affect decision making. Participants described ‘huge concerns with living alone’ for example. These individual attitudes to risk may also be influenced by organisational policies or cultural narratives on risk and protection:

“…safety comes before choice, he wanted to stay at home and he’s not safe at home. It is quite a dilemma in its own way.” (CHO8)

### Sub-theme (c) Practice and system context

Everyday practice and characteristics of the wider system also influenced the decision making process. Although the HSCP participants found the vignettes useful and contained most of the information they needed, the process of allocating services was necessarily artificial. They stressed the importance of knowing the person and their circumstances and how this knowledge might lead to different decisions in ‘real life’:

“…if you know the person it would be easier…so I felt at some of the times I was giving them loads of services… . But if you knew the person, you would tailor it to personalise it for them… I would be able to do a good package because knowing the services and knowing her would make it better.” (CHO8)

In practice, HSCPs can try different combinations of service, start with a low number of hours and see what happens. In the exercise, they felt they were sometimes ‘over-providing’ as they did not have this opportunity to ‘test and review’ service levels:

“… from listening to patients talking, sometimes they find it difficult when we go in with too much care. Whereas, if you went in and assessed first of all, say with an hour a day, and then feel your way and then they may be more receptive to increasing it to one and a half to two hours a day.” (CHO3)
“We had a case where the family complained that there was too much service, too many people.” (CHO2)

However, scarcity of services is a more typical problem:

“And I think if I’m honest, I don’t know would two of those [cases] have got any services, they may just have been waitlisted.” (CHO3 comment on the first two case types – relatively low needs)

Access to quality services was also considered in decision making; not just geographic access but staff availability, for example the shortage of home care workers in some areas; and staff training, whether staff have a sufficient level of training and skills to provide the necessary care:

“…you’ve got quite tight geographic areas where actually we can deliver home support quite economically because we don’t have that far to travel.” (CHO9)
“If these home helps have specific dementia training, it can bring on a huge improvement in clients. …you’ve got your dementia support worker, who’ve got an awareness of dementia or education on it. So it’s amazing how they can change, personalities can change with somebody who knows what they’re doing.” (CHO3)

There was variable understanding among participants of dementia cognitive therapies, reablement, referral processes for some services, and the distinction between roles such as dementia adviser or case manager. This affected whether these services were allocated, or not, for some case types.

This myriad of factors influenced the decisions HSCPs made on the type and amount of services to provide to the different dementia cases in the exercise. There was a continuous weighing up of the different contextual factors in order to decide what services should be provided. The effect of the budget constraint was that certain contextual factors became much more important and this is considered in more detail in Table 5 below.

## Theme 4: Decision-making process

The framework in Figure 2 illustrates how information on whose needs are being considered, what needs are evident and the personal, cultural and system contexts are all weighed up in the decision-making process. However, this is simply an organising framework to illustrate the decision-making process, as the discussions did not occur in the organised, linear manner portrayed in the diagram. The design of the workshops specifically prompted participants to consider why they reduced the services they did in the BC scenario and what priorities they were trying to address:

“Well no, if they were living alone with no supports, that obviously would be the priority.” (CHO3)
“Anywhere where there was, you know, where the carer burden was low, I reduced all the in-home respite and things like that because they seemed to be managing, and scraped back to the basics. (CHO2)

Table 3– Table 5 present a more detailed analysis of the decision-making process, taking into account the service response that was typically arrived at following consideration of all the information.

**Table 3.** Synthesis of themes 1 and 5 in both conditions to identify heuristics.

Theme 1: Whose Needs?			
No constraint	Allocation outcomes	Budget constraint	Allocation outcomes
Needs for all are considered: • Needs specific to the person with dementia • Needs specific to the carer • Needs of the dyad/family	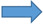 Designed with the separate needs of both in mind as well as supporting the dyad and wider family	• Personal care needs of the person with dementia prioritised • Carer’s needs that will help them to continue caring are prioritised • Carer-specific needs not prioritised	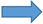 ◦ Services prioritised which will meet the direct needs of the person with dementia *as well as* providing indirect support to the carer ◦ Minimal response to the carer-specific needs
		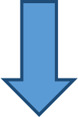 **Heuristic 1**: with constrained resources, supports for the person with dementia are prioritised. Supports for the carer are focused on maintaining their ability to continue caring for the person with dementia.	

**Table 4.** Synthesis of themes 2 and 5 in both conditions to identify heuristics.

Theme 2: What Needs?			
No constraint	Allocation outcomes	Budget constraint	Allocation outcomes
• Needs considered in a holistic way for both the person and the carer - physical., medical and social/psychological 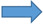 • Emphasis on intervening early to develop care relationships, maintain abilities and to prevent premature deterioration or crisis	**Services**: Full array of services and supports allocated to meet the identified separate needs of person and carer based on preferences of person and carer **Functions**: Emphasis on all functions: discipline- specific assessment for clinical needs; making referrals and linking the person and carer with a range of services; regular and proactive monitoring; and coordination of the range of responses **Roles**: Full range of HSCP disciplines involved	• Personal care ‘meeting basic needs’ prioritised above everything else • Essential clinical needs and/or clinical needs that might increase risk are prioritised 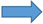 • Carer burden prioritised • Early intervention and prevention not prioritised Social/psychological needs not prioritised	**Services**: Home care prioritised - hours for personal care only to meet the direct needs of the person and indirectly support the carer. Referral to mental health services and other clinical services only prioritised if identified needs in these areas. Small amount of low cost psychosocial support allocated – e.g. support group for both, or one week respite per annum for carer to prevent burn out. **Functions**: All functions scaled back. Emphasis on monitoring to address safety issues and risk. No proactive or preventive referrals. Less coordination as fewer services. **Roles**: Narrower range of HSCPs involved –just for needs identified in case vignettes. No early intervention. 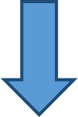
		**Heuristic 2:** with constrained resources, personal care and clinical needs of the person, and carer burden are prioritised (H2). **Heuristic 3:** with constrained resources, proactive or preventive care for the person with dementia and the carer, and psychosocial needs for both are not prioritised (H3).	

HSCP, health and social care professionals.

**Table 5.** Synthesis of theme 3 and 5 in both conditions to identify heuristics.

**Theme 3: Context**			
No constraint	Allocation outcomes	Budget constraint	Allocation outcomes
Multiple variables considered and effort made to take the personal and family context into account in allocating service response 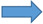 Understanding and knowledge of the range of potential services and supports (clinical, psychological and social)	Service and other responses take into account personal and family context and preferences. Wider knowledge of potential services and supports enables the HSCP to tailor the service response to the individual needs of clients.	• People living alone are prioritised • People with one carer of advanced age and/or with significant physical or mental health difficulties are prioritised 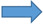 • People with poor living conditions are prioritised	**Services**: Services which fill several functions are prioritised e.g. day care – ‘supervision’ for the day, meal provided and social contact. Preferences not given priority **Functions**: All functions scaled back. Emphasis on monitoring to address safety issues and risk. **Roles**: Narrower range of HSCPs involved –just for needs identified in case vignettes. No early intervention. 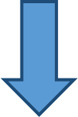
		**Heuristic 4:** With constrained resources a limited number of personal context factors are considered – those that pose a risk and those that most directly relate to the person and/or impinge on the ability of the carer to provide support are to the fore. **Heuristic 5:** Need as much knowledge about the person and their circumstances as possible, to tailor the optimum support package for this person at this point in time and to avoid under- or over-provision.	

HSCP, health and social care professionals.

## Theme 5: Allocation outcomes

The outcome of the decision making process was largely quantitative; the service types and the amount of each which was allocated to each case type, captured using spreadsheets. These data will be reported separately. However, analysis of the discussions revealed that HSCPs did not just think about the service types and roles described in Table 2 when trying to meet needs. They also described functions associated with service provision. In practice, allocating services/resources to respond to need is not as straightforward as selecting from services on a service list. Three ‘allocation outcomes’ were identified in the qualitative data:

1. *Service types* – while these included all the services on the service list, participants tended to focus on home care hours and day care provision;
2. *Functions* – specific functions were identified by participants that were not on the service list. Some of these are implicit in specific services but HSCPs talked in detail about these functions as ways of meeting needs:
  a. assessment;
  b. monitoring and supervision;
  c. information giving;
  d. support (emotional and practical);
  e. ‘linking in’/referral on to other services; and
  f. co-ordination (including key worker activity and case management)
3. *Roles* – while a number of roles were included on the service list participants tended to allocate roles in two ways. Firstly, as a response to a specific need, for example referring the person to a dietician if there were nutrition or swallowing difficulties. Secondly, linking roles to functions such as the PHN for monitoring, or the dementia advisor for information giving (although this latter function was mentioned in relation to several roles).

## Analysis of decision making when budget constraints are introduced

In order to explore the effect of the resource constraint on decision-making, further examination of the data across the themes was conducted. This was to identify patterns in the decision making of the HSCPs when faced with the budget constraint. Table 3 combines the findings from theme 1 with theme 5 under the two conditions. Thus, for theme 1 *whose needs*, in the unconstrained scenario the needs of the person with dementia, the carer and the dyad/family are considered separately and together and the resultant response has elements that respond to the needs of each party. However, when the budget constraint is introduced, the focus is on the care needs of the person with dementia and on addressing burden for the carer. Thus, services for the carer are provided with the primary aim of supporting them to continue in their caring role, not to address other needs they might have. The heuristic identified from this analysis stated that; *with constrained resources, supports for the person with dementia are prioritised. Supports for the carer are focused on maintaining their ability to continue caring for the person with dementia* (Heuristic 1 (H1)).

In Table 4, the data from theme 2 *what needs* are combined with theme 5 *allocation outcomes* under the two conditions. In the NBC scenario, participants welcomed the opportunity to meet the wider needs of the person and the carer within a psychosocial model. They also emphasised the importance of being proactive and adopting preventive approaches. When the budget constraint was introduced, HSCPs tended to prioritise personal care and clinical needs. Two heuristics were identified. Firstly, *with constrained resources, personal care and clinical needs of the person, and carer burden are prioritised* (H2); and secondly, *with constrained resources, proactive or preventive care for the person with dementia and the carer, and psychosocial needs for both are not prioritised* (H3).

In Table 5, the data from theme 3 context are combined with theme 5 *allocation outcomes* under the two conditions. The use of contextual knowledge, of the person/family, culture/values and the wider service context are critical in shaping the service response. The lack of a constraint allowed the participants to take full account of context and to tailor the supports to suit the context. The effect of the constraint was to narrow the range of contextual factors considered to those that might pose or exacerbate risk. The resulting heuristic is framed as: *with constrained resources, a limited number of personal context factors are considered – those which pose a risk and those which most directly relate to the person and/or impinge on the ability of the carer to provide support are to the fore* (H4). However, participants recognised that they need to use their knowledge of the person and family to shape *how* they provide support and not to go in with too much, too soon but to use the options of trying different supports and scaling up or down as needs change: *need as much knowledge about the person and their circumstances as possible to tailor the optimum support package for this person at this point in time and to avoid under- or over-provision* (H5).

## Discussion

Decision making on resource allocation in healthcare in Ireland has long been described as being opaque and ad hoc, with poorly documented processes ( Oireachtas Committee on the Future of Healthcare, 2017; Tussing, 1985). This study presents novel empirical data on resource allocation decision making in community-based dementia care. Micro-level (individual care) and meso-level (local budgetary level) decision making is examined in two scenarios: with and without a budget constraint. The five themes identified in the analysis of the qualitative data from the study illustrate the range of information HSCPs are taking into account as they make these decisions. The framework shows how this information is used to make decisions and reveals the importance of contexts: personal, cultural and system, in shaping these decisions.

Five decision rules or ‘heuristics’ were identified – essentially short cuts or ‘rules of thumb’ which are unwritten but which emerge in the contrast between the two conditions of budget constraint and no constraint. These heuristics provide a new and greater insight into how decisions around dementia care are made and how budget constraints influence decisions. The notion of a ‘threshold’ for care seemed to be implicit in the decision-making schema, i.e. a level of need whereby the person is allocated certain services (or not). Some CHOs (but not all) had explicit criteria for the allocation of home care services, but these did not seem to be widely known among all community based HSCPs in a given area. The use of heuristics did not necessarily lead to poor decision-making, since experiential knowledge gained through practice and communication with the client is very important, but it does raise the possibility of bias and potential horizontal and vertical inequity in the allocation process. However, if the process relied only on rules, the opposite effect might be seen, whereby equity could be realised but at a cost of being able to respond more flexibly to need as it arose in different circumstances.

Understanding resource allocation processes is important in the current budgetary context in health of increasing demands on limited resources. The resultant emphasis on ‘doing more with less’ ( Burke *et al.*, 2014) presents a particular challenge for dementia services which have been largely under-developed in Ireland. The data from this study elucidates the effect of constraints on decision-making and care and in doing so, highlights the gap between policy rhetoric and implementation when there are limited resources, but also points to ways in which we might maximise current resources.

Freedom from a budget constraint allowed HSCPs to think about the needs of the person, carer and family separately (H1) and to consider a much broader range of services, many of which they would not normally consider. They also took a more expansive view on what needs should be considered, with a particular emphasis on adopting a proactive approach to preventing or limiting potential future need (H3). In the budget constraint scenario, participants described having to cut ‘all the good things’ or cut ‘quality of life’ as they had to prioritise personal care needs at a minimum (H2). Decision makers were often conflicted about the focus on personal care needs, recognising that people with dementia were likely to have multiple social and psychological needs, not all of which could be addressed by simply providing additional hours of nursing or home care.

The effect of the budget constraint overall was to narrow all considerations, limiting the type of needs considered and the range of services and supports, resulting in a largely reactive, less personalised system of care (H2, H3). Services for carers were reduced to the minimum that would support them to continue caring, rather than services that might also enhance their quality of life (H1). These effects are counter to stated policy goals of personalised care, prevention, maintaining ability and supporting carers ( Department of Health, 2012; Department of Health, 2013; Department of Health, 2014).

Despite the variation across participant disciplines, sites and workshops, participants were relatively homogenous in their decision-making and capable of making complex resource allocation decisions, providing insight into the importance of dialogue, deliberation and transparency in the allocation process. However, they found the decision-making process in the constrained scenario at times frustrating, also raising ethical concerns. The frustration arose from the effect of the constraint in reducing the possibility of a proactive preventive approach, which they felt would be beneficial and potentially cost saving. The imperative to ‘reduce services’ to people who really needed support, in order to meet the budget constraint was a source of ethical concern which has been reported in other studies ( Scott *et al.*, 2019).

The range of needs represented in the six case types provided an opportunity to examine decision making across the course of dementia. Previous studies have typically focused on cases at the boundary of care between the community and residential care, i.e. cases 5 and 6 in this study ( Tucker *et al.*, 2016). The inclusion of cases with a lower level of need and those at an earlier stage of dementia prompted discussion of the importance of being proactive in supporting the person to maintain their abilities over the course of the disease. This was a particularly strong feature driving resource allocation in the NBC scenario. Concern for people with lower level needs was maintained even when budget constraints were introduced, suggesting that fairness played some role in the decision-making process ( Scott *et al.*, 2019). Participants’ allocated services to all dementia case types, even if they had a low level of relative need, although they acknowledged that this is not what would happen in practice, as cases with low level needs would typically be ‘waitlisted’. This tension about balancing spend on high need individuals and their immediate care versus spend on services which may have benefits later in the care pathway exists in many care systems, and is resolved too often by ignoring or underplaying prevention ( Livingston *et al.*, 2020)

Responding to needs was not as straightforward as matching a service to a need (Theme 5). HSCPs described a number of functions performed by an array of services and they often allocated services in order to meet multiple needs. For example, day care provision can meet needs for social contact, meaningful activities, nutrition and monitoring by a health professional as well as respite for the carer. Contacts with different health professionals and services are potentially opportunities to provide information, emotional support and signposting and many HSCPs do this informally. This practice is analogous to the ‘ making every contact count’ model. Rather than a potentially unhelpful dichotomy between clinical and social care, the provision of good quality responsive care, provided by trained professionals operating flexibly and to the maximum of their role, can potentially address a number of needs in one engagement. Participants were aware that not all services on the list were available to people with dementia, but they recognized that this was the reality of current resource allocation in Ireland. However, these gaps represented missed opportunities for their clients as noted by one participant from CHO2 when she remarked; *‘These services sound wonderful, if only we had a fraction of them’*.

Methodologically, the BoC approach offers the potential to incorporate a mix of existing local data, research findings and experienced practitioner judgements into the decision-making process in a way that is transparent to participants and exposes its key assumptions to critical debate ( Tucker *et al.,* 2013). In that way it sheds much needed light on how local practitioners think about the resource allocation process for different people, in different circumstances. This can be of enormous benefit to policy-makers in estimating budgetary needs at key transition points for people with dementia. A core objective of earlier BoC studies is the identification of people whose care needs could be met in more than one setting (people ‘on the margins of care’), with the alternatives typically involving a choice between community and residential provision. Importantly, the framework does not prescribe a particular course of action, but rather encourages service planners to look beyond existing service delivery, informing decision making and service redesign and acting as an aid to thinking about resource allocation ( Phillips & Bana e Costa, 2007; Tucker *et al.,* 2013, Tucker *et al.,* 2015). The BoC approach does not presume or prescribe a normative allocation of resources in favour of one form of care over another. That ultimately depends on the consideration of both costs and consequences.

What do these findings mean for resource allocation in practice? They demonstrate the importance of an integrated and co-produced assessment process that has a consistent way of describing a person’s preferences, living circumstances and availability of informal care, as well as their professionally assessed clinical and medical needs. Many countries have moved towards a single assessment strategy, including; England ( Challis *et al.*, 2010); Wales ( Wales Government, 2014); and Canada ( Hogeveen *et al.*, 2017); and a Single Assessment Tool (SAT) is being implemented in Ireland ( HSE, 2017).

A deep understanding of the person’s priorities and needs is a pre-requisite for personalisation ( Wilberforce *et al.*, 2017), ensuring that the person gets the right amount of support to maintain their abilities and autonomy, rather than providing inappropriate support ( Keogh *et al.*, 2018a). Such an approach would be relevant to all aspects of the dementia care pathway from diagnosis and initial support services to more intensive care in the later stages, perhaps involving case management ( Challis *et al.*, 2002; Challis, 2003). If we are serious about implementing personalised services, HSCPs need to have access to, and understand the role and function of, a wider array of potential services and supports, such as in a social prescribing model ( Brandling & House, 2008). Thus, cases that are earlier in the course of dementia may still be ‘waitlisted’ in terms of formal health services but could be referred to an array of psychosocial supports such as support groups, Alzheimer cafes and community activities. This requires resourcing for a wider array of such supports nationally, which is still at very low levels ( Keogh *et al.*, 2020). There has been too much conservatism in the menu of services and supports that are currently available for people with dementia. This paper has shown a willingness among HSCPs to draw on psychosocial supports if these services are available.

A more holistic assessment process with a good knowledge of the person and careful consideration of a wide array of responses requires allocated time on the part of HSCPs. The use of heuristics was not just because of the resource constraint. Heuristics are short cuts or rules of thumb when a large amount of information needs to be considered in time-pressured situations. What could be considered a ‘conflation’ of needs with services, could alternatively, be a highly internalised decision making process on the part of the HSCP where, through clinical experience and long practice, they make a quick assessment based on the available information and identify the most appropriate existing service that meets multiple needs – *‘this person needs day care’*. Greater time and engagement in a shared decision making process with the person and family may result in a more responsive and tailored care plan ( Howard *et al.*, 2019; Keogh *et al.*, 2018b). Heuristics are understandable in the face of budget constraints and time pressures, but too often they are bounded by existing models of provision and therefore can never capture the uniqueness and complexity of individual circumstances.

## Conclusion

The qualitative data examined in this paper provides both micro- and meso-level exploration of resource allocation decision-making among HSCPs for people with dementia living at home in Ireland. Participants in the study were asked to make decisions on resource allocation for six different dementia case types, representing just under half of all people with dementia in Ireland. These decisions were made under constrained budget scenarios and unconstrained budget scenarios. Freedom from a budget constraint allowed HSCPs to consider a broader range of services and to take a more expansive view of need, with a particular emphasis on proactive, preventative responses. The effect of the budget constraint was to narrow all options, curtailing the range of service and supports provided, resulting in a largely reactive, less personalised system of care. The budget constraint led to the adoption of heuristic rules, which reinforced existing provision and supported a focus on core personal care needs over more psychosocial models of care. However, even with budget constraints, HSCPs provided some level of support to people with relatively low levels of need. The critical question may not lie between the influence of constrained or unconstrained budgets, since all care systems operate under a degree of scarcity and constraint. Rather, it is what level of additional resource in dementia care would facilitate a wider range and quality of such services in the community.

### Limitations

Although the HSCP participants found the vignettes useful and that they contained most of the information they needed, the process of allocating services was necessarily artificial. An important theme for this group was how they might make different decisions in ‘real life’ particularly the importance of knowing the person and their circumstances and the effect of incremental rather than single step resource allocation. This has important implications for processes such as assessment and care planning. The absence of physicians is a potential limitation, though the exercise did include the vast majority the de facto day-to-day decision-makers in relation to the allocation of services and supports, including people with dementia and family carers, both of whom will be the subject of a separate paper. Physicians are largely absent from resource allocation in social care in Ireland. They certainly would, more than likely, have brought a more clinical orientation to the decision-making process, conveying both advantages and disadvantages while doing so. Nevertheless, further research exploring the values and beliefs of stakeholders within and outside the decision-making process (including the barriers and facilitators to service change) might be particularly instructive.

## Data availability

It was not possible to remove identifying details sufficiently from the data in this study (focus group and interview transcripts), to ensure the anonymity of the research participants. As a result, this data cannot be made available publicly. However, data from the current research can be made available for further research upon reasonable request if the research team is assured participants’ anonymity can be protected. To access the data, please contact the corresponding author ( fiona.keogh@nuigalway.ie). Researchers will be asked to provide a short proposal on how the data will be used before access is granted. All of the vignettes and the COREQ checklist are available in the Zenodo data repository at this link: https://doi.org/10.5281/zenodo.4309313.
