# Supplementary material for: Resource allocation decision-making in dementia care with and without budget constraints: a qualitative analysis
Source: HRB Open Res. 2020 Dec 14;3:69. Originally published 2020 Sep 28. [Version 2] doi: 10.12688/hrbopenres.13147.2 (PMC7808053; doi:10.12688/hrbopenres.13147.2)
Supplement: Peer review history

# Peer review: Reviewer response for version 2

**DOI:** 10.21956/hrbopenres.14338.r28544

**Published:** 2021-01-13

**Authors:** Deirdre O'Donnell

**Affiliations:** 1 School of Nursing, Midwifery and Health Systems, University College Dublin (UCD), Dublin, Ireland

Thanks for the opportunity to review the amendments to the paper. I'm happy to approve it for indexing now and wish the authors well in their future work in this very interesting and important field.

Is the work clearly and accurately presented and does it cite the current literature?

Yes

If applicable, is the statistical analysis and its interpretation appropriate?

Not applicable

Are all the source data underlying the results available to ensure full reproducibility?

Partly

Is the study design appropriate and is the work technically sound?

Yes

Are the conclusions drawn adequately supported by the results?

Yes

Are sufficient details of methods and analysis provided to allow replication by others?

Yes

Reviewer Expertise:

Health and social care of older people (health policy, evidence synthesis and implementation).

I confirm that I have read this submission and believe that I have an appropriate level of expertise to confirm that it is of an acceptable scientific standard.

# Peer review: Reviewer response for version 2

**DOI:** 10.21956/hrbopenres.14338.r28545

**Published:** 2020-12-17

**Authors:** Michael Donnelly

**Affiliations:** 1 School of Medicine, Dentistry and Biomedical Sciences, Queen's University Belfast, Belfast, UK

Reads very well. Thanks.

Is the work clearly and accurately presented and does it cite the current literature?

Yes

If applicable, is the statistical analysis and its interpretation appropriate?

Not applicable

Are all the source data underlying the results available to ensure full reproducibility?

Partly

Is the study design appropriate and is the work technically sound?

Yes

Are the conclusions drawn adequately supported by the results?

Yes

Are sufficient details of methods and analysis provided to allow replication by others?

Yes

Reviewer Expertise:

Evaluation of HSC.

I confirm that I have read this submission and believe that I have an appropriate level of expertise to confirm that it is of an acceptable scientific standard.

# Peer review: Reviewer response for version 1

**DOI:** 10.21956/hrbopenres.14263.r28182

**Published:** 2020-11-19

**Authors:** Deirdre O'Donnell

**Affiliations:** 1 School of Nursing, Midwifery and Health Systems, University College Dublin (UCD), Dublin, Ireland

Thank you for the opportunity to review this highly innovative and relevant article which focuses on decision-making of health and social care professional regarding community resource allocation to support people with dementia living at home.

**Introduction**

 The article is clearly structured and the study is placed within a broader context. In the introduction there is a reference to ‘balance of care methodology’ as having been widely applied to the exploration of care needs of people with dementia. While recognising the limit on word count – the paper could benefit from greater explanation of how resource allocation when viewed through this pragmatic lens may result in an over-reliance on residential care – particularly when relative costs are considered over and above person-centred outcomes. This provides a significant context for the innovative approach adopted for this study which examines ‘micro’ decision-making on resource allocation across a continuum of dementia (varying stages of disease progression) and with a particular focus on community or home care which includes consideration of ‘informal’ family care.

**Methods**

 There is sufficient detail in the description of the methods to facilitate replication. The workshop participants included multi-disciplinary health and social care professionals who had ‘direct experience of working with people with dementia or allocating services to people with dementia’. Were all of the HSCPS who participated working in a community or integrated care team for older people? Some more description of the settings in which they were operating would have been useful here – did they have experience of working collaboratively together prior to the workshop or were they recruited from separate locations? It would appear from the presentation of results that they are recruited from across different CHOs – perhaps some elaboration would be useful here. Furthermore, I note that there are no physicians included in the workshops. This should be discussed as a potential limitation to the study.

 The nominal group technique was very well described with an innovative use of case types and vignettes which provided an excellent simulation for context-based discussion. The inclusion of a sample vignette and case description was a very welcome addition and facilitates a good insight into the data generation process.

 Some greater clarity as to the selection process for the 14 participants who were recruited for interview would be useful. Furthermore, it is unclear in the data reporting which source of data is being referenced (workshop or interview). The methods section (or discussion) might benefit from some reflection upon the qualitative distinction between the two sources of data.

**Results**

 The results are clearly presented using a thematic structure which is very well supported by an illustrative conceptual model which explains the fluidity between heuristics and the influencing factors on decision-making. The discussion of the themes are supported by reference to individual participants who are identified by their CHO area. This does not adequately facilitate an audit trail to the participant. Was there more than one participant from each CHO? If so how are they distinguished from each other by these identifier codes? Perhaps, if issues of confidentiality don’t prohibit it, the discipline of the participant could be identified as this would allow for some understanding of the disciplinary context for their position?

**Discussion**

 The discussion of the findings provided a comprehensive synthesis of the data with integration into the existing knowledge base. Would it be possible to reflect upon disciplinary perspective of the participants that was afforded by the multi-disciplinary nature of the workshops?

 There is a very interesting discussion of the artificial set-up of the workshops and how this removed the participants from the real-world context or the personal knowledge that they might have of the older person and/or their family carer. Could the authors also reflect upon the underlying premise of the set-up which pre-supposes that the list of services provided to the participants are available for allocation? Did the participants comment upon this and whether this was a reflection of their experiences? Furthermore, was it possible for participants to add more services to the list or to create services? If so this would provide an interesting discussion point regarding the contextual boundaries for decision-making in terms of the range of and quality of support services that should be provided by additional resources.

 It is noted that participants exceeded their budget limitation when allocating resources to some older people and that they argued it was not possible for them to stay under their limit. Was it possible for them to shift budget allocation across cases? For example, could they move an underspend from one case to another? Would this have provided some insight into how their perception of need influences their rationing of resources?

 Thank you again for the opportunity to review this article which makes a significant contribution to an understanding of the decision-making of HSCPs with regard to resource allocation. I look forward to reading more project outputs, particularly the perspectives of older people themselves which will undoubtedly contribute to this very important topic.

Is the work clearly and accurately presented and does it cite the current literature?

Yes

If applicable, is the statistical analysis and its interpretation appropriate?

Not applicable

Are all the source data underlying the results available to ensure full reproducibility?

Partly

Is the study design appropriate and is the work technically sound?

Yes

Are the conclusions drawn adequately supported by the results?

Yes

Are sufficient details of methods and analysis provided to allow replication by others?

Yes

Reviewer Expertise:

Health and social care of older people (health policy, evidence synthesis and implementation).

I confirm that I have read this submission and believe that I have an appropriate level of expertise to confirm that it is of an acceptable scientific standard, however I have significant reservations, as outlined above.

# Response

**Published:** 2020-12-07

**Authors:** Fiona Keogh

**Affiliations:** NUI Galway, Ireland

Thank you Deirdre for reviewing our paper and for the detailed observations and comments which have been very helpful. We have made the following changes to the paper in response:

- A paragraph on the BoC approach has been added to the Discussion
- Further detail on the HSCP participants and their working environment has been provided in the Methods.
- The absence of physicians from the workshops is discussed as a limitation.
- Further clarity is provided on the selection of participants for follow-up interviews and the source of data reported in the paper.
- Detail providing clarity on the participants in the workshops has been provided in the Method. Given the group nature of the discussions it was not possible to attribute comments to individuals or disciplines.
- Comment has been added to the Discussion on the absence of marked differences between disciplines in the decisions made.
- Comment has been added to the Method and Discussion on service availability in Ireland and the reaction of participants to gaps in services.
- Detail has been added to the Method to explain the way in which participants could allocate services.
-  The distribution of resource across case types will be covered in a future paper, that will show how participants react to need across case types, especially when budget constraints  apply. Further discussion is beyond the scope of the current paper.

Many thanks for your considered comments which have enhanced the paper. We also appreciate your positive comments.

# Peer review: Reviewer response for version 1

**DOI:** 10.21956/hrbopenres.14263.r28006

**Published:** 2020-11-17

**Authors:** Michael Donnelly

**Affiliations:** 1 School of Medicine, Dentistry and Biomedical Sciences, Queen's University Belfast, Belfast, UK

This paper reports the first part of a mixed methods study that investigates the important topic of professional decision-making, with specific reference to decision-making about the use and allocation of resources in the context of caring for people with dementia and their family or informal carers. The important topic has received sparse empirical attention in the RoI despite government policy statements and commitments and, so, this study addresses a key knowledge gap that provides valuable insights for policy makers, service planners, managers, and HSC professionals. The bottom-up, grounded approach to the development of materials (e.g. vignettes) and conduct of study processes (e.g. nominal group ‘rounds’) resonated well with the experiences of the HSCP participants. Nevertheless, the authors ask us (appropriately so) to remain mindful of the difference between decision making in ‘real life’ dementia care contexts and the decision making scenarios (with and without budgetary constraints) that were studied during the workshop exercises. The results of the study illustrate clearly, the complex and layered nature of care professional decision-making and, arguably, the way in which consideration of budgets constrain and shift focus away from stated policy goals (eg personalised and individualised care decisions). The final report or paper is likely to be even more illuminating when the results of the quantitative and qualitative components of the study are mixed or triangulated. I have noted below a number of points about the paper for consideration by the authors.

 Consider expanding the results section of the Abstract (and, if necessary, reducing the methods section). Add a few sentences for the benefit of non-Irish readers about the health system in relation to dementia care and resource allocation decisions. The reporting of the study followed the 32-item ‘good practice’ methodological checklist. I have not conducted a point-by-point inspection of the study against this checklist and it may be reassuring for readers if the completed COREQ checklist was included as a supplementary file or an appendix. The paper also follows the first steps in the guideline or checklist for reporting the methodology of a mixed-methods study. Presumably, there was some variation in decision making processes and outcomes across or between, for example, 20 different types of HSCPs, 4 regions and 5 workshops? Did this variation point to any new insights about how to arrive at good decisions for people with dementia and their family? Is there a need to explain why the five themes are evidenced by quotations from the same (four) participants – does this finding have significance for the interpretation of the results. Did the 6 vignettes represent around half of the types of cases of people with dementia and, if so, what are the types of cases that were not considered in the decision-making exercises or workshops and what is the significance of this aspect of the study procedures for the results of the study? What was the influence of rural-urban variation on decision making? The results seem to suggest that HSCPs have as part of their decision-making schema the idea of a ‘threshold’ (as well as several context-related professional ‘rules of thumb’). Discuss whether or not this self-conceived or handed-down (via organisational policies or procedures) notion of threshold is significant for dementia care decision-making. The paper is framed in the Introduction as adopting a balance of care approach – consider revisiting the BoC approach in the Discussion and explicate further its significance for decision making about dementia care in RoI. Consider expanding further on the meaning of the results of the study in terms of what changes (if any) to policy and practice should be considered. Finally, the way in which the researchers involved ‘stakeholders’ such as people with dementia, their family carers, advocacy organisations and HS managers is commendable.

Is the work clearly and accurately presented and does it cite the current literature?

Yes

If applicable, is the statistical analysis and its interpretation appropriate?

Not applicable

Are all the source data underlying the results available to ensure full reproducibility?

Partly

Is the study design appropriate and is the work technically sound?

Yes

Are the conclusions drawn adequately supported by the results?

Yes

Are sufficient details of methods and analysis provided to allow replication by others?

Yes

Reviewer Expertise:

Evaluation of HSC.

I confirm that I have read this submission and believe that I have an appropriate level of expertise to confirm that it is of an acceptable scientific standard.

# Response

**Published:** 2020-12-07

**Authors:** Fiona Keogh

**Affiliations:** NUI Galway, Ireland

Thank you Michael for taking the time to review the paper and for your detailed comments. Your comments and suggestions are most helpful and we have made the following changes to the paper:

- changes to the abstract have been made
- the COREQ checklist has been uploaded to the data repository indicated in the paper
- to address the issue of variation across participants and regions additional text has been included in the Discussion.
- Quotations for the themes were drawn from a range of participants in the workshops. However, this was not clear enough in the text. It was not possible to identify every contribution from each participant in the workshops - these were group discussions. Additional text has been added to the method section to provide clarification.
- in relation to the query about how representative the vignettes were, we consulted a wide range of resources in developing the case types. However, the vignettes could not cover all possible case types, especially the more complex cases. There is a risk that in concentrating on the most prevalent types, some less populated, but nonetheless important (and potentially costly) sub-groups will be overlooked. And a few participants did raise the issue of the need to consider each individual’s unique situation, suggesting the potential for a more mixed methods approach and/or greater service user involvement. That said, the case types used in the paper represent just under half of all potential cases, making the results applicable to many people with dementia. Moreover, the key focus in the paper is on the decision-making process itself which will resonate with the vast majority of people with dementia in the country. The process, and the deliberations embedded within, will largely remain constant across all case types.
- The concept of threshold in decision making has been added to the Discussion
- The balance of care approach and its significance for decision making on dementia care in Ireland is explored in more detail through the addition of a new paragraph in the Discussion.
- While some changes to policy and practice have been considered in this paper, further issues will be addressed in subsequent papers form this study.

The authors appreciate the time taken to read and comment on the paper and also the positive comments made.
